# Supplementary material for: Learning curve and surgical outcome of robotic assisted colorectal surgery with ERAS program
Source: Sci Rep. 2022 Nov 29;12:20566. doi: 10.1038/s41598-022-24665-w (PMC9709162; doi:10.1038/s41598-022-24665-w)
Supplement: Supplementary file 2 — Supplementary Information 2. [file 41598_2022_24665_MOESM2_ESM.zip › RAL Raw data-Ñ[▒K/112/OP N_ñw╝╨░O▒Kñσ.pdf]

W83 / W83

# 臺中榮民總醫院
## 手術室護理記錄

[redacted]

### 基本資料

- 室別： 開刀房-17室，手術科別： CRS，手術類別： 常規，日期： 2021/09/15
- 進入時間： 0805 ； 麻醉開始時間： 0835 ； Time Out時間： 1015 ； 劃刀時間： 1016 ； 手術結束時間： 1507 送出時間： 1535 ；
- 麻醉方式： GE
  診斷： Malignant tumor of rectum 8 cm FAV
  手術名稱： Robot Assisted Colectomy(L)
  手術局部用藥： nil
  術前預防性抗生素：
    - 抗生素名稱一： Veterin 1GM IVD/由麻醉護理人員給予
    - 時間： 0925
    - 上次簽章: 歐真勤
  ，追加預防性抗生素：
    - 抗生素名稱一： Veterin 1GM IVD由麻醉護理師給予
    - 時間： 1325
    - 上次簽章: 王明盈
- 手術醫師： 林俊佘，陳瑋夫
- 麻醉醫師： VS 陳永銘
-
    - 刷手護士A ： 王明盈/黃?翔，時間： 0805，刷手護士B ： 謝佩吟，時間： 0805，接替刷手一： 劉雅汶/黃?翔，時間： 1200，接替刷手二： 劉雅汶/呂昭蓉，時間： 1500
    - 流動護士A ： 歐真勤，時間： 0805，接替流動一： 王明盈，時間： 1200，接替流動二： 李思瑩，時間： 1500

---

### 皮膚狀況

A-V Shunt： 無
術前： 完整
記錄者： 上次簽章: 歐真勤
術後： 完整
記錄者： 上次簽章: 李思瑩

---

### 手術準備

手術臥式 單一手術臥式

-
    - 手術臥式： 截石臥式
    - 使用附件： 溫毯 38 ℃，頭圈，腳架
    - 皮膚準備： 10%Aq Betadine，Hibiscrub，2% Chlorhexidine
    - 電燒： 使用電燒: 電極片位置： 其它： 導電軟墊，雙極電燒，記錄者： 上次簽章: 歐真勤

止血帶T： 無

---

### 植入物

- 植入物名稱/號碼/數量/部位： 達文西sure form 45mmx3釘/rectum

，記錄者： 上次簽章: 王明盈

---

### 標本送檢

- 細菌培養 無
- 細胞學 無
- 冰凍切片 無
- 病理標本
  組織名稱一：rectum
  ，
  組織名稱二：IAM Root

,
組織名稱三： distal cutend
，更多組織名稱
  - 組織名稱四： IMV Root

- 截肢 無

標本交付 標本： 3 袋，標本： 0 盒，標本： 1 瓶，細胞學： 0 管，Culture Tube： 0 管，被交付醫師： CR 陳瑋夫，

- 記錄者： 上次簽章: 王明盈
- 時間： 1455

---

## 傷口術後敷料

傷口敷料： 傷口敷料： 無

- 請於圖上標示部位：

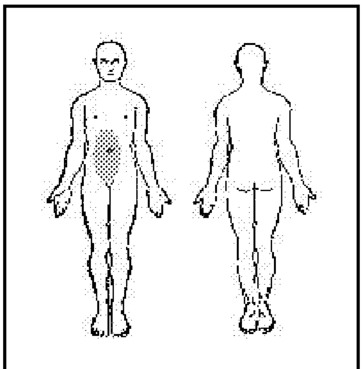

腹部trocar洞

- 傷口敷料： mepore

---

## 導管

| 導管 | 天數 | 導管資訊 | 插入日期 | 插入時間 | 更換日期 | 拔除日期 | 拔除時間 | 拔除原因 | 預計拔除/更換 | 預計拔除/更換日期 |
|---|---|---|---|---|---|---|---|---|---|---|
| Hemo Vac(1) | 1 | 號碼:15 Fr.; 部位:骨盆腔; | 2021/09/15 | 1410 | | | | | | |
| CVC(1) | 1 | 號碼:8 Fr; 深度:15cm; 部位:jugular (右); 象限:RU; | 2021/09/15 | 0900 | | | | | | |
| Sump NG Tube(1) | 1 | 號碼:16 Fr.; 深度:60cm; 部位:胃; | 2021/09/15 | 0905 | | | | | | |
| Foley(1) | 1 | 號碼:16 Fr.; 部位:膀胱; | 2021/09/15 | 0910 | | | | | | |
| A-line (1) | 1 | 號碼:20 Ga; 部位:左手; | 2021/09/15 | 0850 | | | | | | |

---

## 用物計數

- 手術中出血量： minimal c.c.
- 手術中尿量： 300 c.c.
- 
  - 其它： 無

用物計數： 多切口同時縫合

- 1.器械： 正確，2.縫針： 正確，3.刀片： 正確，4.紗布： 正確，5.腹部墊： 未使用，6.紗球： 未使用，7.PEANUTS ： 未使用，8.紗條： 未使用，9.絲帶： 未使用，10.針頭： 未使用，11.Vessel loop： 未使用，12.Boots： 未使用，13.Jelco： 未使用，14.棉條： 未使用，
  - 第一次：
    - 刷手護士 ： 劉雅汶/黃?翔
    - 流動護士： 上次簽章: 王明盈
    - 時間： 1420

  ,
  - 第二次：
    - 刷手護士 ： 劉雅汶/黃?翔
    - 流動護士： 上次簽章: 王明盈
    - 時間： 1435

,

- 第三次：
  - 刷手護士 ： 劉雅汶/黃?翔
  - 流動護士： 上次簽章: 王明盈
  - 時間： 1450

## 備註

備註： davinci0度scopy:NO2

- 記錄者： 上次簽章: 歐真勤
- 時間： 0805

填寫護理人員 李思瑩

正式摘要

W83 / W83

doctorRank:B
